# Multivariate Identification of Functional Neural Networks Underpinning Humorous Movie Viewing

**DOI:** 10.3389/fpsyg.2020.547353

**Published:** 2021-02-09

**Authors:** Fa-Hsuan Lin, Hsin-Ju Lee, Wen-Jui Kuo, Iiro P. Jääskeläinen

**Affiliations:** ^1^Department of Medical Biophysics, University of Toronto, Toronto, ON, Canada; ^2^Sunnybrook Research Institute, Toronto, ON, Canada; ^3^Department of Neuroscience and Biomedical Engineering, Aalto University School of Science, Espoo, Finland; ^4^Institute of Neuroscience, National Yang-Ming University, Taipei, Taiwan; ^5^Brain Research Center, National Yang-Ming University, Taipei, Taiwan; ^6^International Laboratory of Social Neurobiology, Institute of Cognitive Neuroscience, National Research University Higher School of Economics, Moscow, Russia

**Keywords:** functional connectivity, comedy, movie, networks, humor

## Abstract

While univariate functional magnetic resonance imaging (fMRI) data analysis methods have been utilized successfully to map brain areas associated with cognitive and emotional functions during viewing of naturalistic stimuli such as movies, multivariate methods might provide the means to study how brain structures act in concert as networks during free viewing of movie clips. Here, to achieve this, we generalized the partial least squares (PLS) analysis, based on correlations between voxels, experimental conditions, and behavioral measures, to identify large-scale neuronal networks activated during the first time and repeated watching of three ∼5-min comedy clips. We identified networks that were similarly activated across subjects during free viewing of the movies, including the ones associated with self-rated experienced humorousness that were composed of the frontal, parietal, and temporal areas acting in concert. In conclusion, the PLS method seems to be well suited for the joint analysis of multi-subject neuroimaging and behavioral data to quantify a functionally relevant brain network activity without the need for explicit temporal models.

## Introduction

Converging evidence suggests that complex naturalistic scenes and stimuli elicit neuronal responses more reliably than simplified stimuli in conventional laboratory experiments (Mechler et al., 1998; Yao et al., 2007; Belitski et al., 2008). Accordingly, there is an emerging trend of using naturalistic stimuli, including movies, TV shows, and musical pieces, to study human brain function (Hasson et al., 2004; Jaaskelainen et al., 2008; Alluri et al., 2012; for review, see Hasson et al., 2010). Such experimental techniques have been suggested to be more appropriate to probe the neuronal responses related to complex cognitive processes common in our daily life, such as narrative comprehension (Wilson et al., 2008; Regev et al., 2013) and movie watching (Hasson et al., 2004, 2008b; Jaaskelainen et al., 2008). Note that the sense of humor is a unique feature of human social life and likely involves complex cognitive processes. Accordingly, the neural substrates responsible for the sense of humor have been investigated using naturalistic stimuli, including cartoons (Moran et al., 2004; Azim et al., 2005; Bartolo et al., 2006; Wild et al., 2006; Samson et al., 2008, 2009), short verbal passages (Ozawa et al., 2000; Goel and Dolan, 2001; Uekermann et al., 2006; Watson et al., 2007; Bekinschtein et al., 2011), and movie clips (Moran et al., 2004; Neely et al., 2012).

Typically, the waveforms from distributed brain areas are found to be correlated during naturalistic stimulation, corroborating the hypothesis that cognitive functions are mapped at the level of multi-focal neural systems (Mesulam, 1990). Distributed brain areas have been reported to be functionally related to humor processing, including the inferior frontal gyrus (Goel and Dolan, 2001; Mobbs et al., 2003; Azim et al., 2005; Bartolo et al., 2006; Watson et al., 2007; Bekinschtein et al., 2011), middle frontal gyrus (MFG) (Azim et al., 2005; Samson et al., 2008), superior frontal gyrus (Bekinschtein et al., 2011), middle temporal gyrus (MTG) (Moran et al., 2004; Bartolo et al., 2006), temporal pole (Mobbs et al., 2003), supplementary motor area (Mobbs et al., 2003), inferior parietal lobule (Ozawa et al., 2000), and subcortical structures (Mobbs et al., 2003). From this, it follows that the analysis of functional magnetic resonance imaging (fMRI) data collected under naturalistic stimulation would optimally consider the nature of diffusive functional areas and their interactions.

Functional magnetic resonance imaging data obtained during naturalistic stimuli are typically analyzed by calculating the inter-subject correlation (ISC), which is done by first aligning the brains of individual subjects and then calculating Pearson correlations between the hemodynamic activity time courses for each voxel (Hasson et al., 2004). This makes it possible to inspect the degree of similarity in how the brains of individual subjects respond to common naturalistic stimuli. Such an analysis method does not require explicit temporal model on the dynamics of the imaging data, which is often difficult to describe when continuous naturalistic stimuli are presented. Another analysis method is representational similarity analysis (RSA) (Kriegeskorte et al., 2008), which explores the correlation between brain responses and behavioral recordings or stimulus features. However, both ISC and RSA as univariate approaches have clearly one limitation: it ignores the potential spatial correlation among brain areas, which is likely involved in processing of naturalistic complex stimuli as argued above. To circumvent this challenge, multivariate analysis methods, such as the independent component analysis (ICA), have been developed and applied to conventional fMRI experiments (for review, see Calhoun et al., 2009). There have been pioneering studies using ICA to disclose functional areas activated during movie viewing (Zeki et al., 2003; Bartels and Zeki, 2004; Jaaskelainen et al., 2008; Malinen and Hari, 2011; Calhoun and Pearlson, 2012; Lahnakoski et al., 2012; Mantini et al., 2012). These methods decomposed the gigantic four-dimensional neuroimaging data directly. Physiologically sensible networks were then identified when the time courses associated with (spatial) independent components were significantly correlated between subjects (Bartels and Zeki, 2004; Jaaskelainen et al., 2008) and when the time course associated with the spatial independent component and carefully annotated stimulus feature were correlated (Lahnakoski et al., 2012). However, after multivariate decomposition, the relative contributions of conditions and interactions between conditions are typically unknown. This poses the difficulty in providing explicit models to test hypotheses using conventional approaches.

Instead of direct multivariate decomposition of the four-dimensional neuroimaging data using either the principle component analysis (PCA) or ICA (Erhardt et al., 2011; Kauttonen et al., 2015), in the present study, we set forth to use partial least squares (PLS), to analyze the correlation between neuroimaging data, experimental conditions, and behavioral measures (McIntosh et al., 1996; McIntosh and Lobaugh, 2004) and to identify large-scale neuronal networks (for review and tutorial, see Krishnan et al., 2011). To the best of our knowledge, PLS has not been applied to reveal the neural underpinnings and the behavioral correlates of the dynamic processing of complex naturalistic stimuli.

In this study, we developed a PLS analysis method to reveal the brain networks with synchronized neural or behavioral dynamics when processing ecologically relevant stimuli. Then, we used this method to discover the neurofunctional underpinnings of the experienced humor during free viewing of comedy clips. Specifically, the proposed PLS analysis decomposes an effect space constructed by correlating either the experimental contrasts of interest or a behavioral measurement of experienced humorousness over time. Such decomposition discloses and compares networks underpinning the cognitive processes. Different from the direct decomposition of the neuroimaging data, PLS analyzes the correlation between neuroimaging data and hypotheses or behavioral measurements. This formulation allows faster calculation and easier interpretation of the decomposed spatial patterns in relation to experimental designs and behavioral responses. Importantly, the estimated spatial structure in each decomposed component naturally considers the spatial correlation in the analysis. Based on the PLS framework, we found spatial structures showing similar blood oxygen level-dependent (BOLD) fMRI signal dynamics across subjects during free viewing of humorous movie clips. We also elucidated networks that had temporal waveforms closely related to subjective rating of humorousness over time. PLS results were compared with the conventional univariate analysis results to show that the proposed multivariate analysis is more sensitive in identifying neural correlates to the processing of complex naturalistic stimuli. This multivariate network analysis approach may be applied to analyze multi-subject neuroimaging data without explicit temporal models.

## Materials and Methods

### Subjects

Twenty healthy volunteers (age range 20–25; 12 females; normal or corrected to normal vision) participated in the study. A written informed consent was obtained from each subject prior to participation. The study was run in accordance with the guidelines of the Declaration of Helsinki, and ethics approval was obtained from the Ethics Committee of the National Yang-Ming University prior to commencing the research. Parts of data were used in our recent univariate analysis on neural correlates to sense of humor (Jaaskelainen et al., 2016).

### Stimuli and MRI Acquisition

The subjects were presented with three movie clips (4′33″, 5′17″, and 5′08″ in duration) twice, in an order that was randomized across subjects, during 3T (Tim Trio, Siemens, Erlangen, Germany) fMRI [echo-planar imaging (EPI); repetition time/echo time (TR/TE) = 2,000/30 ms, field of view (FOV) = 220 × 220 mm, matrix = 64 × 64, slice thickness = 4 mm, flip angle = 90°]. The movie clips were taken from comedy-genre movies “The Circus” and “City Lights,” directed by Charles Chaplin and produced by Charles Chaplin Productions in 1928 and 1931, respectively. There were pauses of 2 min between the runs in order to wash out the effects of the preceding clip. Prior to the fMRI, a high-resolution *T*_1_-weighted anatomical (MPRAGE sequence, TR/TI/TE/flip = 2,530 ms/1,100 ms/3.49 ms/7°, partition thickness = 1.33 mm, matrix = 256 × 256, 128 partitions, FOV = 21 cm × 21 cm) scans were obtained from each subject.

### Self-Reported Humor Ratings

Immediately after the fMRI session, the subjects were re-shown, without advance warning, the three comedy movie clips and were asked to rate, on a Likert scale running from 1 to 10, once every 15 s, the degree of amusement that they experienced in the first viewing of the movies. A rating of 1 corresponded to lack of experienced humor, and a rating of 10 corresponded to a high degree of experienced humor. We asked participants to recall the degree of humorousness in the first viewing outside the MRI scanner in order to avoid the interruption of naturalistic viewing. In our previous studies (Jaaskelainen et al., 2008; Nummenmaa et al., 2012), it was shown that re-viewing the movie clip actually serves as a robust cue for recall of experienced emotions. In fact, in our previous analysis (Jaaskelainen et al., 2016), it was found that ratings on the degree of humorousness in the first and second viewings of movie clips were highly similar. These online ratings also matched the recalled ratings in different participants, and the ratings obtained with the instruction to recall the first viewing correlated more closely with rating during the first vs. second online viewing (Jaaskelainen et al., 2016). These subjective rating waveforms were SINC interpolated to match the duration of the fMRI measurements. Our previous analysis on these self-reported humor ratings revealed no significant differences among three movies (Jaaskelainen et al., 2016).

### Functional Magnetic Resonance Imaging Preprocessing

As described in our previous study (Jaaskelainen et al., 2016), EPI data were first pre-processed by applying motion correction, slice-timing correction, and spatial smoothing (3D isotropic Gaussian kernel with the full width at half maximum = 10 mm). Subsequently, individual-subject EPI data were first spatially registered to individual anatomical data using the 12-parameter affine transformation as implemented in FSL^1^ and then transformed to cortical surfaces using a template (“fsaverage” in FreeSurfer; version 5.1)^2^. Note that subsequent PLS analyzed EPI data on the cortical surfaces.

### Partial Least Squares Analyses

Here, we describe two versions of the PLS analysis to disclose spatial structures showing correlated dynamics across subjects (inter-subject PLS) and correlated temporal waveforms in neuroimaging time series and behavioral measurement (behavioral PLS). PLS is a multivariate analysis approach aimed at revealing brain activation maps and the associated experiment factors based on the partial correlation matrix between the neuroimaging data and experimental designs or behavioral measures (McIntosh et al., 1996).

### Inter-Subject Partial Least Squares

To perform the PLS analysis, we first created a cross-correlation matrix **M**. In inter-subject PLS, each entry **M** is the inner product between the fMRI time series from two subjects at one cortical location. Specifically, we first calculated the inner product between a pair of subjects (*m*_*i*_ and *m*_*j*_; *i* ≠ *j*) at a specific brain location *s* in an experimental condition with index *c*.

(1) $${\text{M}}_{p,s}^{c}=\left\langle {\text{D}}_{:,S}^{m_{i},c},{\text{D}}_{:,S}^{m_{j},c}\right\rangle$$

where *p* denotes the subject pair [*p* = 1, …, *P*; *P* = 1/2(*n*_*s*_ × (*n*_*s*_ − 1)); *n*_*s*_ is the total number of subject], and ${\text{D}}_{:,S}^{m_{i},c}$ denotes the time series of subject *m*_*i*_ at brain location *s* in condition *c*. In this study, the conditions of our interest were: (1) three movies and (2) two repeated viewings of the clips. Cross-correlation matrices of different conditions were vertically concatenated.

(2) $$\text{M}=\left[\begin{array}{c} {\text{M}}_{•,•}^{c_{1}}\\ \vdots \\ {\text{M}}_{•,•}^{c_{n}} \end{array}\right],$$

where *c*_*i*_’s were indices for conditions (*i* = 1, …, *n*). ${\text{M}}_{•,•}^{c_{1}}$ denotes a sub-matrix **M** for condition *c*_1_ and cross-correlation for all subject pairs and brain locations.

The next step of the PLS analysis is to construct a contrast matrix, which quantitatively encodes the contrast of interest by contrasting between conditions. In our study, we created a contrast matrix **C** encoding both effects of watching individual movies and distinguishing the effect between the first and second viewings of a movie.

      
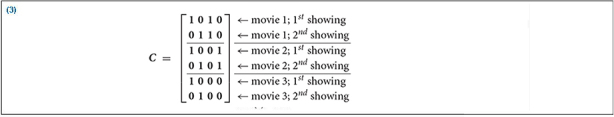


Note that the first two columns of **C** respectively explored effects related to the first and second viewings of movies. The third and fourth columns of **C** respectively explored effects related the first and second movies. No contrast was created to specifically explore the effect related to the third movie, because a linear combination of these four columns of **C** can create this contrast. We cannot reduce the “viewing” contrasts from two to one by ignoring one of the first two columns in **C**, because we also want to reveal the grand average effect. It should also be pointed out that each entry **1** and **0** here represents a column vector of ones of length *P*, the total number of subject pairs. Subsequently, we used singular value decomposition (SVD) to decompose the effect space **E** as the product between **M** and **C**.

$$\text{E}={\text{C}}^{T}\text{M}$$

(4) $$\text{SVD}\left(E\right)={\text{USV}}^{T}$$

Functional networks related to all effects can be found in columns of **U**, **V**, and diagonal entries of **S**. A combination of one column of **U**, corresponding column of **V** and diagonal entry of **S** is called one latent variable (LV). Specifically, the columns of **V** are brain LV’s showing spatial distributions of the salience of each network, whose correlates to experimental conditions can be found in the corresponding columns of **U** (design LV’s), denoting weightings in each contrast specified in columns of **C**. Each diagonal entry of **S** denotes the relative contribution of the corresponding network. More specifically, it is convenient to calculate the product **C** × **U** to reveal the implication of experimental conditions. Each column of **C** × **U** is called one design score. The singular values in **S** quantified the amount of variance by each network.

In this study, we created contrasts for individual movie clips (Eq. 3). It may be speculated that other contrasts, such as a within-task mean-centering one, may yield different results. However, if the new set of contrasts has the same rank and spans as the old set (contrasts of the new set are linear combinations of the old set), the results will be identical. Let us use **C*_*n*_*** to denote a new contrast matrix, which is transformed from **C** by a unitary matrix **T**.

$${\text{C}}_{n}={\text{CT}}^{T}$$

$${\text{E}}_{n}={\text{C}}_{n}^{T}E=\text{TE}$$

(5) $$\text{SVD}\left({\text{E}}_{n}\right)={\text{TUSV}}^{T}={\text{U}}_{n}{\text{SV}}^{T}$$

The design scores related to the new contrast matrix are identical to those related to the old contrast matrix, because **T** is unitary.

(6) $${\text{C}}_{n}{\text{U}}_{n}={\text{CT}}^{T}\mathrm{TU}=\text{CU}$$

Brain LV’s were also identical using either **C** or **C*_*n*_***. Taken together, transformation contrasts with a unitary matrix do not change the PLS analysis.

The statistical significance of each LV was based on an empirical null distribution estimated by permutation. This was done by first randomly shuffling the time series of each subject of each condition and each brain location. Specifically, we used the adjusted amplitude Fourier transform (AAFT) algorithm to ensure that the shuffled time series preserves the linear correlation structure of the original time series (Theiler et al., 1992). Accordingly, the correlation matrix based on shuffled time series was constructed. The same contrast matrix was used to build the effect space, which was then decomposed by SVD to obtain LVs. This procedure was repeated 10,000 times. The significance of an LV was then calculated by the proportion of the singular value in this empirical null distribution exceeding the singular value in the PLS analysis. The statistical significance of each brain LV and design LV was assessed by a bootstrap procedure, where $M_{p,s}^{c}$ was created from randomly selected subjects with replacement. The bootstrap was repeated 10,000 times, and a pseudo-*Z* score was calculated as the ratio between the original brain LV and the standard deviation of bootstrap samples. To control for multiple comparisons, we used the false discovery rate (FDR) approach (Genovese et al., 2002). The active brain areas estimated in each brain LV were automatically clustered and identified using FreeSurfer with a 400-mm^2^ threshold. We summarized these brain areas by reporting local maximal statistics of each cluster, Talairach coordinates of these maximal statistics, the size of the clustered active area, and the anatomical labels for each cluster.

### Behavioral Partial Least Squares

To more directly disclose the network associated with behavioral measurement, we modify the construction the correlation matrix **M**. Specifically, with synchronous behavior waveforms during the course of neuroimaging data acquisition, we can calculate the correlation between these two measurements:

(7) $${\text{M}}_{i,s}^{c}=\left\langle {\text{D}}_{:,S}^{m_{i},c},{\text{B}}_{:}^{m_{i},c}\right\rangle ,$$

where *i* denotes the subject (*i* = 1, …, *n*_*s*_), ${\text{B}}_{:}^{m_{i},c}$ denotes the behavior waveform for subject *m*_*i*_ in condition *c*, and ${\text{D}}_{:,S}^{m_{i},c}$ denotes the time series of the imaging data for subject *m*_*i*_ in condition *c* at brain location *s*. In this study, we recorded the degree of “funniness” using Likert scale (1: the least funny; 10: the most funny) for each movie clip from each subject after the experiment. Importantly, the subjects were asked to recall the degree of funniness of the first viewing of the movie.

After the calculation of the correlation matrix **M**, and effect space **E** was constructed as described in the inter-subject PLS by **E** = **C**^*T*^**M**, where **C** is the design matrix of our interest. SVD was applied to decompose the effect space to disclose the spatial structure of networks and relative loadings of contrasts encoded in **C**.

The outcomes of SVD over **E** consist of left (**U**) and right (**V**) orthonormal matrices and a diagonal matrix (**S**, Eq. 4). Again, the columns of **V** are brain LV’s representing spatial distributions of the salience of each network. The interpretation of each network can be derived from linear combinations of comparisons of experimental conditions in columns of **U** (design LV’s). Each diagonal entry **S** denotes the relative contribution of the corresponding network. The significances of LVs were similarly evaluated by permutation (with shuffled behavioral measurements), and the significances of the salience of brain LVs were quantified by the bootstrap procedure described above. Brain LV’s in the behavioral PLS analysis were also automatically clustered, summarized, and labeled using the same procedure described in inter-subject PLS.

**FIGURE 1:**
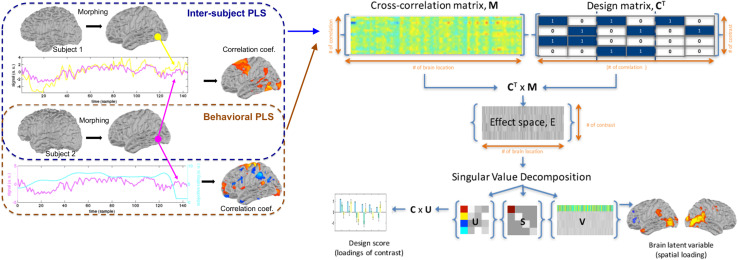
The workflow of inter-subject partial least squares (PLS) and behavioral PLS analyses, including the construction of an cross-correlation matrix **M**, design matrix **C**, the effect space **E**, and the decomposition of the effect space, using singular value decomposition to reveal weightings of different contrasts and the associated spatial distribution of the blood oxygen level-dependent (BOLD) signal.

Figure 1 illustrates the processing flow for both inter-subject PLS and behavioral PLS analyses. All calculations were performed using MATLAB (MathWorks, Natick, MA, United States) on a computational server (Intel Xeon CPU @ 2.2 GHz clock; 64 Gbytes memory; Ubuntu 12.04 release).

### Comparison With Univariate Analysis

To compare results of PLS analysis with those of conventional univariate analysis, we calculated the correlation (ISC) (Hasson et al., 2004) and general linear model (GLM) of the fMRI signals with the subjective rating of the degree of humorousness to compare with inter-subject PLS and behavioral PLS results, respectively. In both univariate analyses, two contrasts were specifically examined: the effect related to viewing all movie clips and the effect related to the difference between the first and second viewings. The statistical significance of ISC was evaluated by a subject-wise permutation test to control the false-positive rate uniformly (Chen et al., 2016). The significance of maps in the GLM of the fMRI signals with the subjective rating of the degree of humorousness was quantified by shuffling the time series of each subject of each condition and each brain location using the AAFT algorithm (Theiler et al., 1992). These permutation tests were performed 10,000 times. The *p*-values were taken as the fraction of the occurrence of the results from the permuted data exceeding those from the unshuffled data. The significance of statistics due to multiple comparisons was adjusted by controlling the FDR (Genovese et al., 2002).

## Results

### Inter-Subject Partial Least Squares

**FIGURE 2:**
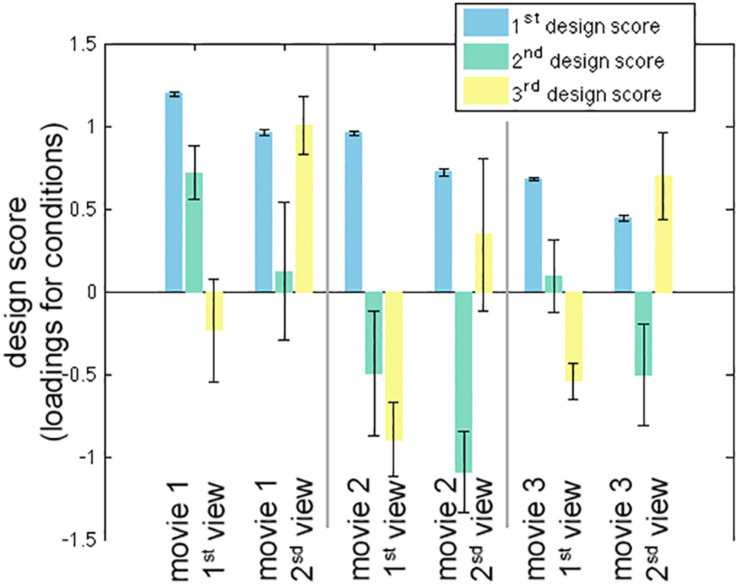
The first three significant design scores of the inter-subject partial least squares (PLS) analysis. The error bars represent the standard deviation estimated from 100 bootstrap steps.

The calculation of one inter-subject PLS analysis took about 1 s. The first three LV’s were found to be statistically significant in the permutation test with 100 iterations (*p* < 0.05). These three LV’s accounted for 98.0, 1.0, and 0.8% of the variance in the effect space. Each SVD decomposition took less than 1 s to complete. The bootstrap procedure with 100 iterations took about 2 min to complete. Figure 2 shows the design scores of the inter-subject PLS. Specifically, the first LV can be considered as an average effect of ISC across three movies and two repetitive viewings. Note that the second viewing in all three movies accounted for less variance of the effect space than the first viewing. This was indicated by the relative strength in the design score of the first LV (Figure 2). The second LV was interpreted as a contrast showing the effect relative among movie clips, because movie 1 had large positive values while movie 2 (and the second viewing of movie 3) had large negative values. The third LV indicated differential sensitivity between the first and second viewings: it described an effect of relatively increased ISC in the second viewing of movies and relatively decreased ISC in the first viewing of movies.

**FIGURE 3:**
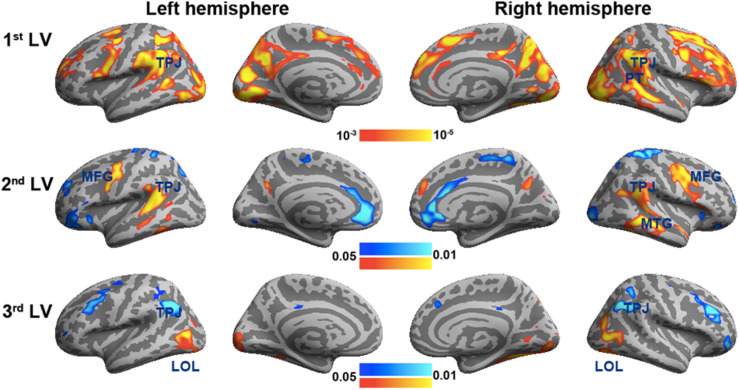
The first three significant brain latent variables of the inter-subject partial least squares (PLS) analysis.

Figure 3 shows the spatial distributions (brain LV’s) of the inter-subject PLS analysis. A significant bi-hemispheric activity in the visual cortex, planum temporale (PT), temporal–parietal junction (TPJ), and lateral frontal lobe (particularly at the right hemisphere) was observed in the first LV. This is consistent with the previous univariate ISC analysis (Tohka et al., 2014). The second LV depicted more clustered areas in the bi-hemispheric TPJ, MFG, and right MTG. Note that the frontal activations in both hemispheres and right supplementary motor areas were found with significant but negative contribution in the second LV, suggesting a stronger effect in movie 2 than in movie 1 in terms of ISC. The third LV delineated areas explaining more ISC in the first viewing than the second viewing (areas with negative values: bi-hemispheric TPJ; more posterior than the TPJ areas in LVs 1 and 2) and the dorsolateral prefrontal cortex (DLPFC) as well as more ISC in the second viewing than the first viewing [areas with positive values: visual areas and lateral occipital lobes (LOLs)]. Table 1 lists the brain areas with significant clusters of activities in the three LVs.

**TABLE 1:** Effects and the related clustered activated brain loci, Talairach coordinates, brain area size, and anatomical labels in ISC-PLS analysis.

Latent variable	Effect interpretation	Hemisphere	Max. value	Area (mm^2^)	*x* (mm)	*y* (mm)	*z* (mm)	Anatomical label
1	Common inter-subject correlation in movie watching	Left	35.0	9,339	–33.6	–41.7	52.2	Superior parietal
			35.0	3,271	–46.7	–49.2	44.5	Supramarginal
			35.0	2,188	–52.4	14.7	15.2	Pars opercularis
			35.0	1,638	–6.1	1.9	55.0	Superior frontal
			35.0	503	–47.5	–53.8	11.0	Bank of the superior temporal sulcus
			34.0	1,767	–23.1	50.4	21.0	Superior frontal
		Right	35.0	3,599	8.4	–58.7	37.7	Precuneus
			35.0	10,057	44.8	1.5	35.8	Precentral
			35.0	5,150	52.5	–33.1	–4.4	Bank of the superior temporal sulcus
			35.0	5,757	21.1	–78.7	–6.4	Lingual
2	Higher inter-subject correlation in movie 1 watching than in movie 2 watching	Left	5.0	1,164	–63.6	–36.9	9.0	Superior temporal
			5.0	860	–45.0	–4.7	45.1	Precentral
		Right	5.0	2,011	59.2	–42.2	22.7	Supramarginal
			5.0	1,288	65.3	–32.9	–4.7	Middle temporal
			5.0	2,309	40.4	3.1	37.0	Caudal middle frontal
	Higher inter-subject correlation in movie 2 watching than in movie 1 watching	Left	–5.0	926	–8.9	44.5	3.3	Rostral anterior cingulate
			–4.7	798	–25.0	43.2	29.4	Superior frontal
			–4.5	1,152	–31.7	32.2	–7.3	Lateral orbitofrontal
		Right	–5.0	1,287	27.2	–28.1	59.6	Postcentral
			–5.0	1,001	7.8	42.6	–9.0	Medial orbitofrontal
			–5.0	994	29.8	–92.1	0.8	Lateral occipital
			–4.8	489	14.5	–36.9	58.7	Paracentral
3	Higher inter-subject correlation at the second view than at the first view	Left	5.0	1,536	–46.6	–75.7	13.6	Lateral occipital
		Right	5.0	1,302	47.6	–75.5	10.6	Lateral occipital
			5.0	2,214	29.2	–53.6	–9.2	Fusiform
	Higher BOLD–humorousness correlation at the first view than at the second view	Left	–5.0	1,023	–44.7	–62.3	43.1	Inferior parietal
			–4.7	1,080	–42.8	23.5	36.0	Caudal middle frontal
		Right	–5.0	816	53.2	–43.8	36.6	Inferior parietal
			–5.0	1,358	39.0	33.0	28.9	Rostral middle frontal
			–4.9	428	31.9	54.6	–9.6	Pars orbitalis

*ISC, inter-subject correlation; PLS, partial least squares; BOLD, blood oxygen level dependent.*

**FIGURE 4:**
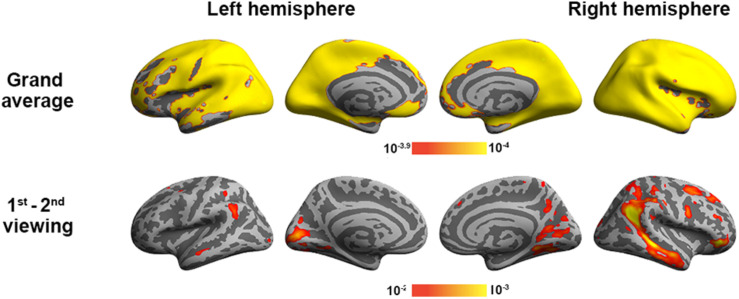
Inter-subject correlation analysis discloses brain areas with fMRI signals synchronized across participants during two repeated movie clip viewings (top row) and the difference between the first and second viewings (bottom row).

To validate the inter-subject PLS analysis, we analyzed the fMRI data by calculating the inter-subject correlated fMRI signal. These correlation maps were compared with the first and third LV’s, representing the overall viewing effect and the difference between the first and second viewings. The whole-brain fMRI signals were significantly synchronized across participants in viewing movies, except part of the left frontal lobe. We found a good match between ISC and inter-subject PLS analyses (Figure 4). Significant areas in the first LV include the visual cortex, PT, TPJ, and lateral frontal lobes. In the third LV, inter-subject PLS revealed that the fMRI time series at bilateral TPJ and right DLPFC were significantly more synchronized in the first viewing than in the second viewing. These areas were also found close to areas identified by the ISC analysis. Note that the contrast was coded in different-polarity PLS (PLS values were different between the second and first viewings, while ISC values were the difference between the first and second viewings).

### Behavioral Partial Least Squares

**FIGURE 5:**
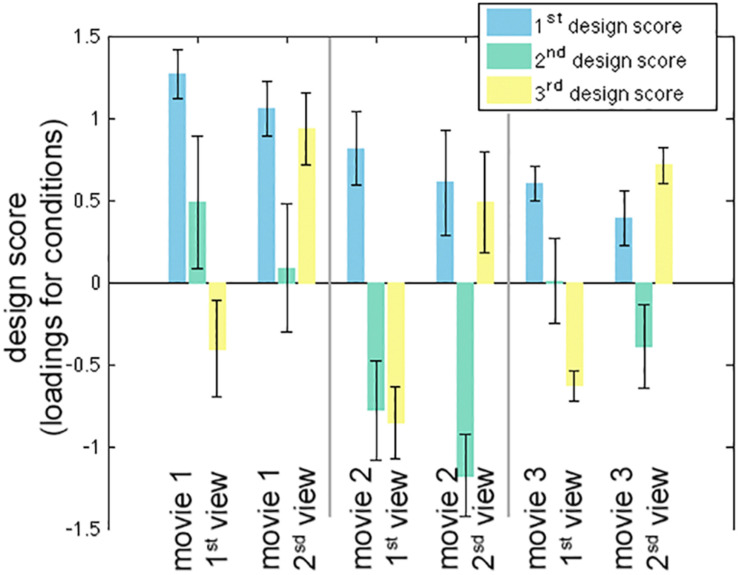
The first three significant design scores of the behavioral partial least squares (PLS) analysis. The error bars represent the standard deviation estimated from 100 bootstrap steps.

The first three LV’s in the behavior-PLS analysis were found significant in the permutation test (*p* < 0.01). These three LV’s accounted for 80.6, 17.3, and 1.6% of the total variance in the effect space. The computational time for each behavior-PLS analysis was similar to that of the inter-subject PLS analysis, because the size of the effect space was the same (about 2 min for 100 bootstraps). Figure 5 shows the first three design scores. From the design score, the first LV represented the average effect on correlating the BOLD signal and subjective rating of the humorousness. Therefore, the corresponding brain LV can be interpreted as brain areas generally sensitive to the humorous content in the movies. The second design score represented a mixture effect of individual movie and repeated showing. Specifically, it represented the relative weighting between the first viewing of movie 1 and the average of two viewings of movie 2 and the second viewing of movie 3, rendering this LV the most difficult to interpret. The third design score indicated the differential effect between the first viewing and the second viewing of the same movie. The polarity of the third design score suggested that the positive values in the third brain LV represented brain areas more sensitive in the second viewing than the first viewing, and brain areas with negative values were more active in the first viewing than the second viewing.

**FIGURE 6:**
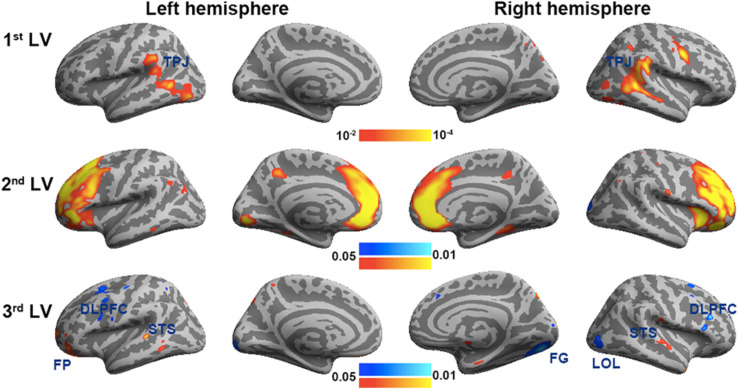
The first three significant brain latent variables of the behavioral partial least squares (PLS) analysis.

Figure 6 shows the three brain LV’s in the behavioral PLS on inflated cortical surfaces. Significantly activated areas in the first LV included the bi-hemispheric TPJ and right MFG. The second brain LV included mostly the left and right frontal areas in both medial and lateral aspects. The third brain LV included both areas with positive and negative values. The areas with positive values were clustered in the superior temporal sulcus (STS) in both hemispheres and left frontal pole (FP), showing a positive correlation between the BOLD signal and subjective rating of humorousness in the second viewing and a negative correlation in the first viewing of movies. Areas with negative values included the superior, middle, and inferior frontal areas; right fusiform gyrus (FG); and right LOL. These areas had a positive correlation between the BOLD signal and subjective rating of humorousness in the first viewing of movies and a negative correlation in the second viewing of the movie clips. Table 2 lists these significant brain area clusters in three LVs.

**TABLE 2:** Effects and the related clustered activated brain loci, Talairach coordinates, brain area size, and anatomical labels in behavior–PLS analysis.

Latent variable	Effect interpretation	Hemisphere	Max. value	Area (mm^2^)	*x* (mm)	*y* (mm)	*z* (mm)	Anatomical label
1	Correlation between BOLD signal and humorousness	Left	9.3	1,115.6	–52.1	–59.7	8.1	Middle temporal
			9.0	374.6	–57.4	–40.9	36.7	Supramarginal
			8.5	444.3	–56.9	–45.6	20.0	Superior temporal
		Right	10.0	584.4	49.0	1.4	34.6	Precentral
			10.0	3,138.4	57.8	–45.3	5.2	Bank of the superior temporal sulcus
2	Higher BOLD–humorousness correlation in movie 1 watching than in movie 2 watching	Left	5.0	10,910.4	–6.6	42.3	–12.8	Medial orbitofrontal
			5.0	681.9	–3.3	–87.8	–3.2	lingual
		Right	5.0	9,891.2	6.6	37.0	13.2	Rostral anterior cingulate
			3.9	465.2	36.4	–40.6	–5.5	Fusiform
3	Higher BOLD–humorousness correlation at the second view than at the first view	Left	4.9	1,024.5	–13.5	34.6	–19.9	Lateral orbitofrontal
	Higher BOLD–humorousness correlation at the first view than at the second view	Left	–3.8	409.4	–21.2	10.9	45.6	Superior frontal
			–4.7	883.4	27.0	–81.3	–3.8	Fusiform

*PLS, partial least squares; BOLD, blood oxygen level dependent.*

**FIGURE 7:**
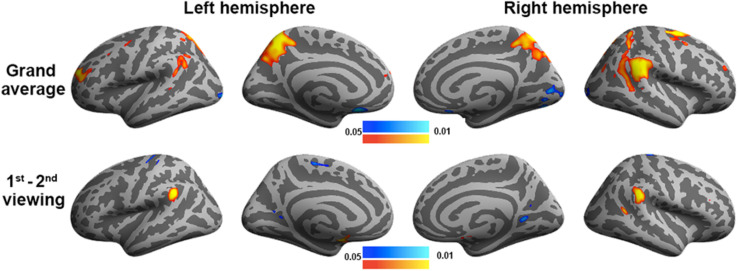
General linear modeling discloses brain areas with fMRI signals correlating to the degree of humorousness in two repeated viewings (top row) and the difference between the first and second viewings (bottom row). Color codes for the *p*-values uncorrected for multiple comparisons.

We also analyzed the fMRI data using GLM to contrast the behavioral PLS analysis (Figure 7). In particular, we examined two contrasts: (1) the grand average of the correlation between movie viewing and the experienced degree of humorousness and (2) the difference in the correlation between movie viewing and the experienced degree of humorousness in the first vs. second viewing. No significant correlations were found by setting the threshold to control the inflation of Type 1 error due to multiple comparisons by controlling the FDR at 0.05. With the use of a lower threshold (uncorrected *p*-value = 0.05), the GLM analysis revealed that the fMRI signal fluctuations at bi-hemispheric visual cortices were negatively correlated with the degree of humorousness, while the fMRI signal fluctuations at the left TPJ, right TPJ, left frontal lobe, left parietal lobe, and right parietal lobe were positively correlated with the degree of humorousness. The left and right TPJs matched between GLM and the first LV in the behavioral PLS analyses. Using FDR to control the error in multiple comparisons, we found that the correlation between fMRI time series and degree of humorousness in the first viewing was not significantly different from that in the second viewing. At a lower threshold (uncorrected *p*-value = 0.05), we found that the correlation between fMRI time series and degree of humorousness in the first viewing was larger at the bi-hemisphere TPJ than that in the second viewing, while the third LV in the behavioral PLS analysis suggested that bi-hemispheric STS and DLPFC show the difference in the behavior–brain correlation between repeated viewings.

## Discussion

In this study, we developed a computationally efficient method to disclose spatial patterns of fMRI dynamics that are similar across subjects during free viewing of movie clips as well as jointly correlated patterns of the brain activity and experienced humorousness. To our knowledge, this is the first PLS-based multivariate analysis method to disclose neural and behavioral correlates to complex naturalistic stimuli processing. This is also the first functional connectivity analysis to reveal networks showing a synchronous activity across subjects and between individual’s brain activity and experienced humorousness. Therefore, the findings of this study hold novelty in both methodological development and cognitive neuroscience. Specifically, inter-subject PLS revealed the visual cortex, TPJ, and right frontal lobes showing strong synchronous BOLD signal time courses across subjects during free viewing of the movie clips (Figure 3). To further pinpoint networks related to humor processing, behavioral PLS suggested that BOLD signals at bi-hemispheric TPJ and dorsal lateral frontal lobes are closely related to the subjective experience of humorousness during free viewing of the comedy clips (Figure 6). Part of these areas matched the results from the univariate ISC analysis (Figure 4) and GLM of fMRI time series and subjective ratings of the degree of humorousness (Figure 7). However, PLS detected more spatially extensive and significantly activated brain areas, such as the contrast between the first and second viewings (Figures 4, 7; the third LVs in both inter-subject PLS and behavioral PLS). Taken together, the proposed PLS method can be a tool to identify brain networks supporting the processing of ecologically relevant stimuli without the need of an explicit temporal model.

Our results corroborated with those of previous studies on movie watching and humor processing. In particular, the first brain LV in inter-subject PLS involving the visual cortex, bi-hemispheric temporal parietal lobes, and right frontal lobe matched the findings from previous studies on movie watching (Hasson et al., 2004, 2008a, 2010; Kauppi et al., 2010; Nummenmaa et al., 2012; Cantlon and Li, 2013). The synchronous activity in these sensory, integrative, and high-order areas across subjects suggested that movie information was perceived in a similar manner. The first brain LV in behavioral PLS revealed a set of brain areas showing correlated dynamics of subjectively experienced humorousness and BOLD signal, including the bi-hemispheric TPJs and right ventral lateral prefrontal cortex. These temporal lobe areas have been previously found to be active when subjects are watching cartoons (Moran et al., 2004; Bartolo et al., 2006).

We included contrasts for individual movies (Eq. 3) in order to provide more degrees of freedom in modeling the correlation between neural activities or between a neural activity and behaviors during viewing different movies. These contrasts increased the dimension of the effect space. Some LVs contrasting between movie clips, such as the second LV in both inter-subject PLS and behavioral PLS, should be interpreted as the stronger/weaker activity when viewing different movies, instead of the neurofunctional representation for a specific movie.

In this study, two movie clips were extracted from the same movie. However, their scenes were quite different. Except for the main character, no character was repeatedly shown in different clips. In examining the design scores in both inter-subject-PLS and behavioral PLS (Figures 2, 5), there was no effect clearly related to two clips except for the same movie. Consequently, we considered this concern to be relatively minor.

One essential aspect of the PLS analysis is that it is a data-driven method capable of quantitatively disclosing interactions between multiple contrasts of interests. In this study, we investigated the selective and common aspects of humor processing between different movie clips and how novelty affects the feelings of mirthfulness when repeatedly showing the same movie clips. While it is possible to encode these contrasts using an explicit model, as in our preliminary findings (Tohka et al., 2014), potential interactions among these effects are, in fact, unknown. On the other hand, we first created an effect space summarizing all effects of our interest. Then the relative weightings of different contrasts were automatically estimated in PLS via a multivariate decomposition (SVD in our case). Accordingly, we found three separate networks accounting for BOLD signal that correlated across subjects: these respectively accounted for different effects: common to three movies (the first), selective to one movie in relation to the other two (the second LV), and the difference between the first and second showing of three movies (the third LV). The behavioral PLS similarly disclosed three networks accounting for the correlation between BOLD signal and subjective rating of humorousness. The capability of decomposing the collection of neuroimaging data across experimental conditions and hypotheses into separate spatially distributed patterns and relative condition/hypothesis weightings exemplified the values of the multivariate analysis.

Partial least squares is computationally efficient due to the fact that multivariate decomposition, PCA in this study, operated on the effect space with much reduced dimensionality via the direct incorporation of hypotheses to neuroimaging data in the construction of the PLS effect space. In this study, if an effect space was not first constructed, one would have had to decompose the fMRI data with up to 150 time points per subjects. In the group PCA/ICA analysis using temporally concatenated data, the temporal dimension can be as large as 3,000 when 20 subjects were recruited. On the contrary, PLS dramatically decreased this dimension to four in both inter-subject PLS and behavioral PLS (the dimension of the effect space). The reduced dimensionality in the PLS improves not only the computational efficiency but also the interpretation of LVs, because they were linear combination of the proposed hypotheses.

Our proposed PLS analysis can be taken as a natural extension of the original PLS method (McIntosh et al., 1996) and a later application of PLS to event-related fMRI data (McIntosh et al., 2004). Earlier versions of PLS need to obtain the event-related responses, which are typically extracted from explicitly given temporal markers corresponding to the onsets of specific stimuli. On the contrary, experiments using complex and naturalistic stimuli typically do not have any temporal markers to extract neural responses time locked to specific events. In this study, we demonstrated how to extend the PLS framework to reveal the brain spatial features that account for the inter-subject correlated brain activity across first and repeated viewing of different movie clips. Our analysis considered the fMRI dynamics over the whole interval of movie viewing without the need to extract any event-related responses.

Inter-subject correlation or fMRI–behavior correlations are required be calculated first in inter-subject PLS or behavioral PLS, respectively. Then, SVD was used to decompose the effect space to reveal related brain and design LVs. This is different from the direct multivariate decomposition of neuroimaging data (Erhardt et al., 2011; Kauttonen et al., 2015). When using ICA, such direct decomposition is computationally intensive. On the contrary, inter-subject PLS and behavioral PLS only need to decompose an effect space of much smaller dimension, because the fMRI time series dimension has been already compressed in ISC or fMRI–behavior correlation calculation. Previously, we have also proposed using ICA to decompose the effect space in PLS analysis (Lin et al., 2003). There was no significant difference between SVD and ICA decomposition in PLS using event-related fMRI data; this question is yet to be answered in experiments using naturalistic stimuli.

The proposed PLS methods had the similarity and difference to inter-subject RSA (IS-RSA) methods (Nummenmaa et al., 2012; Nguyen et al., 2019; van Baar et al., 2019; Chen et al., 2020). Like IS-RSA, the ISC-PLS approach used the fMRI waveforms from pairs of participants to calculate an imaging correlation matrix at each brain location (Eq. 1). Then, IS-RSA evaluates the correspondence between the imaging correlation matrix and behavioral response similarity matrix at each brain location separately. Different from IS-RSA, ISC-PLS does not correlate the imaging coefficient matrix with behaviors. ISC-PLS also differs from IS-RSA by using SVD on the cross-correlation between the imaging correlation matrices and comparisons of experimental conditions across the whole brain to reveal sets of brain areas corresponding to these cross-correlations (Eq. 4). The spatial correlation between brain areas is naturally taken into consideration by the SVD. To reveal the correspondence between behaviors and brain signals, at each brain location, we directly calculated behavior–imaging correlation matrices by cross-correlating between the subjective rating and fMRI signal dynamics (Eq. 7) without calculating their correlation matrices separately as IS-RSA. Then, SVD was applied to the cross-correlation between the behavior–imaging correlation matrices and comparisons of experimental conditions across the whole brain to reveal sets of brain areas corresponding to these behavior–imaging cross-correlations.

In this study, we pre-processed data by spatial smoothing with a kernel of 10-mm width. As various degrees of spatial smoothing affect functional mapping and functional connectivity analysis results, the optimal spatial smoothing remains to be studied systematically in the near future.

In conclusion, PLS, a computationally efficient multivariate analysis method, was adapted to reveal across-subjects consistent networks during free viewing of humorous movie clips as well as across-subjects consistent networks sensitive to subjective ratings of humorousness. This method automatically identified relative weightings of different contrasts and related neuronal networks. Specifically, brain areas sharing common BOLD signal dynamics across subjects when watching movies, selectively in one particular movie related to others, and with differential sensitivity to the first and second viewings of the same movies, were identified. The PLS method also suggested three sets of brain areas showing a close correlation between subject ratings of humorousness and BOLD signal with differential experimental condition sensitivities. Taken together, we believe that PLS is a viable tool in functional network analysis when continuous, complex, and naturalistic stimuli are used in neuroimaging experiments.

## Data Availability Statement

The raw data supporting the conclusions of this article will be made available by the authors, without undue reservation.

## Ethics Statement

The studies involving human participants were reviewed and approved by the National Yang-Ming University. The patients/participants provided their written informed consent to participate in this study.

## Author Contributions

F-HL, W-JK, and IPJ designed the experiment. H-JL collected the data. F-HL and H-JL analyzed the data. F-HL, H-JL, W-JK, and IPJ wrote the manuscript. All authors contributed to the article and approved the submitted version.

## Conflict of Interest

The authors declare that the research was conducted in the absence of any commercial or financial relationships that could be construed as a potential conflict of interest.

## Footnotes

1 http://www.fmrib.ox.ac.uk/fsl/

2 http://surfer.nmr.mgh.harvard.edu
